# Occupation-related factors affecting the health of migrants working during the COVID-19 pandemic – a qualitative study in Norway

**DOI:** 10.1186/s12939-023-02013-2

**Published:** 2023-10-17

**Authors:** Pierina Benavente, Elena Ronda, Esperanza Diaz

**Affiliations:** 1 Pandemic Centre, Department of Global Public Health and Primary Care, Faculty of Medicine, University of Bergen, Bergen, Norway; 2 Department of Community Nursing, Preventive Medicine and Public Health and History of Science, Faculty of Health Sciences, University of Alicante, Alicante, Spain; 3 CIBER for Epidemiology and Public Health (CIBERESP), Health Institute Carlos III, Madrid, Spain

**Keywords:** Occupational health, Migrant workers, Migrant health, International migrants, COVID-19

## Abstract

**Background:**

The effects of the COVID-19 pandemic were more pronounced among migrants than in the majority population and went beyond those directly caused by the virus. Evidence suggests that this overburden is due to complex interactions between individual and structural factors. Some groups of working migrants were in vulnerable positions, overrepresented in essential jobs, under precarious work conditions, and ineligible for social benefits or special COVID-19 economic assistance. This study aimed to explore the experience of migrants working in Norway during the COVID-19 pandemic to gather an in-depth understanding of the pandemic´s impact on their health and well-being, focusing on occupation-related factors.

**Methods:**

In-depth personal interviews with 20 working migrants from different job sectors in Bergen and Oslo were conducted. Recruitment was performed using a purposive sampling method. Thematic analysis was used.

**Results:**

At the workplace level, factors such as pressure to be vaccinated, increased in occupational hazards, and increased structural discrimination negatively impacted migrants’ health. Other factors at the host country context, such as changes in social networks in and out of the workplace and changes in the labour market, also had a negative effect. However, the good Norwegian welfare system positively impacted migrants’ well-being, as they felt financially protected by the system. Increased structural discrimination was the only factor clearly identified as migrant-specific by the participants, but according to them, other factors, such as changes in social networks in and out of the workplace and social benefits in Norway, seemed to have a differential impact on migrants.

**Conclusions:**

Occupational-related factors affected the health and well-being of working migrants during the pandemic. The pressure to get vaccinated and increased structural discrimination in the workplace need to be addressed by Norwegian authorities as it could have legal implications. Further research using intersectional approaches will help identify which factors, besides discrimination, had a differential impact on migrants. This knowledge is crucial to designing policies towards zero discrimination at workplaces and opening dialogue arenas for acknowledging diversity at work.

**Supplementary Information:**

The online version contains supplementary material available at 10.1186/s12939-023-02013-2.

## Introduction

The COVID-19 pandemic was a magnifying glass of pre-existing structural global weaknesses, such as discrimination and social inequalities in terms of food insecurity and access to healthcare and social services [1]. The effects of the COVID-19 disease and the consequences of mitigation measures have been amplified among groups in socially vulnerable situations, and migrants are worldwide overrepresented in COVID-19 statistics. In Norway, a country with a 15% of migrant population, 30–40% of infected individuals were born outside the country throughout the pandemic. This overrepresentation extends to hospitalisations and deaths due to COVID-19 [2–4].

Working migrants are often in especially vulnerable positions as they usually work in what has been defined as essential jobs, including cleaning, farming, construction, and healthcare, among others. Several studies have linked these occupations with COVID-19 infection cases [5, 6]. In addition, many working migrants are employed precariously and thus ineligible for sick leave, social security, or special COVID-19 economic assistance, which increases their vulnerability [7]. Pre-pandemic studies had found a higher risk of work-related health problems, bullying and discrimination at work among migrant workers as compared to the majority population [8, 9]. In Norway, migrants are more often under temporary work contracts, experience higher job insecurity, and report a higher incidence of bullying than the majority population [10]. Furthermore, occupational conditions in Norway partly explain higher sick leave levels among migrants [11].

During the COVID-19 pandemic, several outbreaks in accommodations and workplaces with migrant workers were reported worldwide [12]. However, to the best of our knowledge, the impact of this crisis on worker migrants has not been subject to in-depth study [13]. In Norway, around 400 000 workers reported to be unemployed by April 2020, and working sectors where migrants are overrepresented, such as tourism and transportation, were the most affected during the pandemic [14]. In addition, infectious outbreaks in construction sites, where most workers are migrants, have been reported [15, 16]. Despite this, no studies focusing on migrant workers’ health during the pandemic have been conducted in Norway, as to our understanding.

The pandemic has had consequences for mental and physical health beyond those directly caused by the virus in all populations, like anxiety, depression, and weight gain [13, 17]. It has been suggested that complex interactions of different individual and structural factors play a role in the differential impact of the COVID-19 pandemic on working migrants’ health and well-being [3, 17, 18]. However, there is limited evidence of occupational-related conditions as factors for poor physical and mental health in migrants during the pandemic [8, 9]. Therefore, this study aimed to explore the experiences of migrants working in Norway during the COVID-19 pandemic and to gather an in-depth understanding of its impact on their health and well-being, focusing on occupation-related factors.

## Methods

### Study design and setting

This phenomenological qualitative study was conducted in Bergen and Oslo, Norway, between May and September 2022, following COREQ guidelines [19]. We performed in-depth personal interviews with working migrants from different occupational sectors.

### Study context

Norway has a strong and universal welfare state. This system guarantees financial assistance, social protection, and delivery of services, providing security to all citizens against health and economic shocks [4]. The Norwegian Labour and Welfare Administration (NAV) is the entity responsible for financial and social assistance in Norway. The Norwegian welfare system comprehends several programmes and services, such as family and child welfare, unemployment benefits, social assistance, sick benefits, pensions, and health and care services. All citizens legally registered in Norway could access any of these programmes if they meet specific requirements [20].

Unemployment rates in Norway are low. By the end of 2020, the first year of the COVID-19 pandemic, unemployment increased from 3.7% in 2019 to 4.9%. The more affected groups were mainly those with low levels of education, young people, and migrants outside the European Union [4]. By 2023, levels are back to 3.4% of the labour force being unemployed [21].

### Sampling

Purposive sampling was used to recruit participants from several occupational sectors: construction, transportation, cleaning, healthcare, and other (academics, arts, education, and research). These groups were chosen as they cover a variety of migrant profiles: from different regions, lengths of stay in Norway, reasons for migration, and integration profiles. We classified the participants into three job groups according to their exposure to COVID-19 pandemic: Group 1: construction, transportation, and cleaning workers (mostly essential workers with no direct contact with COVID-19 patients), Group 2: healthcare workers (direct contact with COVID-19 patients), and Group 3: other skilled workers (mostly non-essential workers with the possibility to work remotely).

We recruited nine participants utilising contacts from the Inncovid study, a mixed-method study on migrants during the first wave of the pandemic [22, 23]. Two participants were recruited using networks from non-governmental organisations working with migrants who facilitated contact with potential participants. We also distributed leaflets to these organisations and posted ads in Facebook migrant groups (two participants were recruited with this strategy). The participants also recommended peers, and seven participants were recruited using this snowballing technique.

### Data collection

We performed in-depth interviews following a semi-structured interview guide based on the existing literature on ‘migrants’ and ‘migrant workers’ health during the COVID-19 pandemic [13, 24] (Supplementary material 1).

We piloted this interview with one working migrant, and minor adjustments were made to reduce the interview time.

The participants could choose their preferred language for the interviews, using an interpreter when necessary. All interviews were performed by the first author, nine in English and 10 in Spanish. Only one remote video interpreter was used for an interview performed in Somali. Interviews lasted between 40 and 90 min and were conducted at different places chosen by the participants (their homes, workplaces, or cafes). Three interviews were conducted by Zoom at the request of the participants. After 20 interviews, we reached data saturation, meaning no new information was found in the interviews.

All interviews were audiotaped and transcribed verbatim in their original language (English or Spanish) by the first author. Then the Spanish interviews were translated into English using DeepL and proof checked by the first author. Data were analysed using the thematic analysis framework by Braun and Clark. The six phases proposed by this framework were: familiarising with the data, generating initial codes, searching for themes, reviewing themes, defining and naming themes, and producing the report [25]. Analysis was conducted in NVivo 12 using a mixed approach (inductive and deductive) for coding.

Categories were identified from the data following the causal model in occupational health framework proposed by Benavides et al. [26]. This framework purports that several (individual and structural) components positively or negatively influence the health and well-being of workers. In this model, we can observe three different levels: individual (micro), corporate (meso), and governmental (macro) [26].

## Results

Twenty participants were included, and their sociodemographic characteristics are presented in Table 1. Most of our participants were females (12 of 20) and aged 31 to 40. However, all construction workers were male and less than 30 years of age. Regarding the region of origin, the biggest group came from Southern Europe (5 of 20) following by Eastern Africa (4 of 20).

**Table 1:** Sociodemographic characteristics of the participants

Sex	Age category	Region of origin	Type of work	Years in Norway
M	51–60	Southern Europe	Cleaning	10
M	41–50	South America	Cleaning	11
F	31–40	Western Europe	Cleaning	9
F	41–50	Southern Europe	Transportation	8
F	41–50	Eastern Europe	Transportation	5
M	51–60	Eastern Africa	Transportation	9
M	18–30	Southern Europe	Construction	2
M	18–30	Southern Europe	Construction	2
M	18–30	Eastern Europe	Construction	6
F	41–50	Eastern Europe	Healthcare	14
F	31–40	South America	Healthcare	15
F	31–40	Eastern Africa	Healthcare	12
F	31–40	Eastern Africa	Healthcare	19
M	31–40	Southern Europe	Healthcare	9
F	41–50	South-eastern Asia	Healthcare	9
F	31–40	South-eastern Asia	Other skilled worker	15
F	31–40	Western Europe	Other skilled worker	6
F	41–50	Eastern Europe	Other skilled worker	16
F	31–40	South America	Other skilled worker	1
M	41–50	Eastern Africa	Other skilled worker	11

**Fig. 1:**
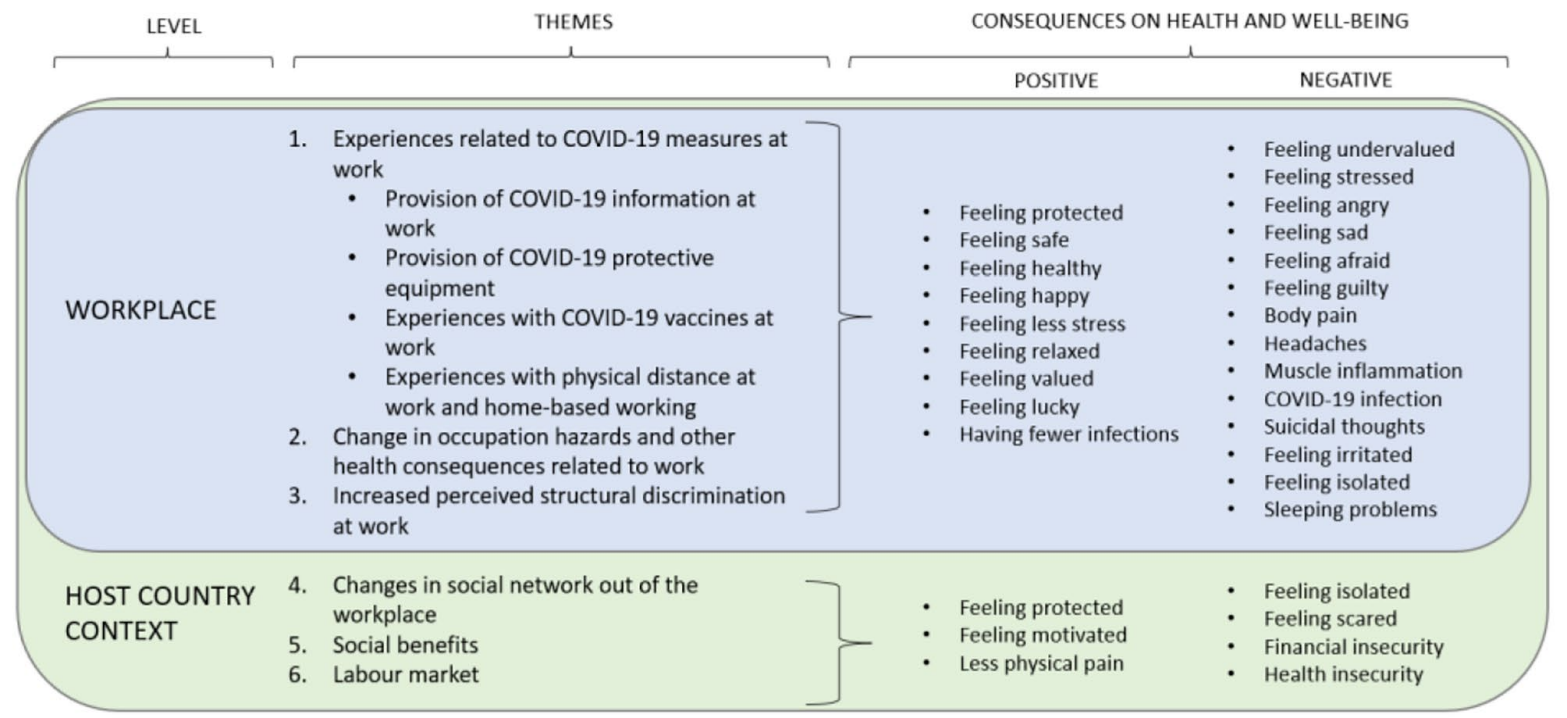
Themes and self-reported consequences in health and well-being

We identified six main themes when analysing the experiences of migrants working in Norway during the COVID-19 pandemic and its impact on their health and well-being. These themes were classified based on the causal model of occupational health framework [26] into two levels: workplace and host country context. At the *workplace* level, we included those factors related to working and employment conditions in which the company or the employer could intervene. The *host country context* level includes the work-related factors beyond the control of the employer or company and those social determinants outside the workplace that affect daily work. In Fig. 1, we show all the themes and the consequences in health and well-being reported by the participants.

### Workplace level

#### Theme 1: experiences related to COVID-19 measures at work

Most participants mentioned the factors below related to their experiences with the COVID-19 measures at work.

##### Provision of COVID-19 information at work

The participants explained that most companies provided them with at least basic advice and information about COVID-19 disease. Several companies provided up-to-date information on an ongoing basis from official sources and adjusted measures at work to protect the workers’ health and well-being. Although most of the time, updated information positively affected health and well-being, some participants mentioned that sometimes it was unclear and confusing.

*The company gave information, they gave information, they sent a lot of communications and a lot of rules; they took a lot of time to decide, a lot! (…) they communicated all the time by messages, by SMS, all the time. We have an intranet, (…) and there they also post a lot of messages, and they communicate constantly (…) I was, well a bit stressed yes, I mean, let’s see, I didn’t have it clear anymore. (Transportation worker from Southern Europe)*

Freelancers or workers assigned to various locations reported that they had to comply with different measures according to the specific workplace, which created confusion.

##### Provision of COVID-19 protective equipment

All participants stated that, when needed, their companies provided protective equipment to avoid COVID-19 infection (antibacterial gel, masks, gloves if applicable), and most of the time, this made them feel safe and protected. However, participants working with groups in vulnerable situations, such as children or older adults (primarily from Groups 1 and 2), found it challenging to use masks when interacting with those people. They mentioned it was difficult to connect or talk to them when wearing masks.

*Uh there was a point where we were wearing masks but we at a some point we just couldn’t (…) Kids need (facial) expressions. (Other skilled worker from Asia)*

##### Experiences with COVID-19 vaccines at work

When the vaccine was available to the public, most workplaces recommended the participants get it. However, some of the healthcare workers experienced that companies or supervisors pressured them to get vaccinated. This pressure negatively impacted their well-being. Participants also stated that in some cases, it was an indirect pressure.

*There was not the question do you want to get the vaccine? It’s like when do you get the vaccine? (…) that was the content of our conversation. There was no question of do you will you get it? (Other skilled worker from Western Europe)*

In other cases, employers put direct pressure on workers to get vaccinated.

*(…) people who wanted to get the first vaccination signed up (…). But a colleague fell ill just the day before receiving the vaccine, so that vaccine could not be missed and so they (employers) put pressure on me to get it. But I didn’t sign up on that list (…) I told them I don’t want to, (…). They said: No! it won’t go to waste, you have to get it, we can’t find someone else (Healthcare worker from Southern Europe)*.

##### Experiences with physical distance at work and home-based working

All participants stated that physical contact with co-workers and clients was reduced in different ways during the pandemic. Most reported that physical work meetings were changed to digital ones, and some participants from groups 2 and 3 reported working remotely. For most participants in all groups, having less social contact with colleagues and clients negatively impacted their mental health, although they knew this could help them avoid getting infected at the same time. In addition, it was a very challenging period for those working with persons in vulnerable situations, and some reported breaking COVID-19 rules for the sake of those they worked for.

*Even at times I took a risk, we met in a school when it was forbidden that nobody should come but the teacher, the principal was so nice she said X please, let us help this family because there were four and they were new in Norway. We cannot just ignore them, why don’t you come? (Other skilled worker from Eastern Africa)*

Participants did not mention whether this factor differed between migrants and the majority population. However, recent migrants in Norway reported that it could have been more challenging for them as they had fewer opportunities to meet people.

*(…), there weren’t even like those passive spaces where you accidentally meet people or accidentally interact with strangers and that’s also cool and create friendships, no, no, no, no. (Other skilled worker from South America)*

Participants with children stated that working at home and helping the children with homeschooling was very challenging, and this affected the quality of their work. Other participants mentioned the positive aspects of homeworking, such as not commuting (and thus not being exposed to the virus), being able to eat healthier and doing exercises.

#### Theme 2: change in occupation hazards and other health consequences related to work

Most participants reported exposure to psychosocial hazards due to the lack of support, increased working hours and new tasks during the pandemic. Some reported feeling worthless as they found themselves performing tasks that were not specific to their role. This negatively impacted their mental health.

*I felt that I was no longer a professional person that is working, I am a person that is only getting people to talk about COVID, to help them washing their hands, hygienically, I mean I felt like wow my profession went down to a very low level (…) (Healthcare worker from South America)*.

Construction workers reported working more hours and feeling exploited during the pandemic, which made them feel stressed and angry. These participants believe the exploitation was related to being migrants.

*It was more pressure on COVID, because we had… they took so many jobs that we didn’t have actually manpower to do it. And then, then it came a lot of pressure and stress for that. (Construction worker from Eastern Europe)*

Cancelling holidays due to COVID-19 restrictions was also mentioned as a factor affecting participants’ mental health during the pandemic.

The transportation workers reported that several workers at their companies were temporarily laid off for some months because not many drivers were needed for public transportation.

Some participants mentioned that physical hazards increased due to the pressure or urge to complete tasks.

*(…) if a patient is too heavy, or if he or she needs more assistance from two or three assistants, we had to go to the patient as a group as two or three (nurses), but then Corona (COVID-19) started, and they limited the personnel. (…) So, I got back pain, muscle inflammation. (Healthcare worker from Eastern Africa)*

At the same time, other participants stated getting COVID-19 at work.

*(…) all of us in the company, we were like 6 or 8 workers, we all got COVID. There was (only) one person working for two weeks because she was the only one who tested negative for COVID. So, of course, that was a big, big problem. (Transportation worker from Southern Europe)*

Participants did not mention whether the increase in occupational hazards differed between migrants and the majority population in Norway. However, most participants from Group 1 did mention that migrants were overrepresented in their companies.

On the other side, some participants mentioned positive changes in their health and well-being:

Most transportation workers reported experiencing fewer psychosocial and physical hazards due to decreased use of public transport.

*Less stress at work. A lot less stress. When I was driving, I didn’t have to stop all the time and look out for any passengers. And then we didn’t have to charge either, of course. So, we were more relaxed driving, (…). (Transportation worker from Southern Europe)*

Moreover, for some participants working with migrant groups who were heavily hit by the pandemic, mental health improved because their jobs were more appreciated by peers than before the pandemic.

*(…) working with minority patients is not like the big focus of the hospital because of… it is minorities. You know, it’s very few patients in the hospital. So because of the COVID it makes my work… it’s more (…) important!. (Healthcare worker from Asia)*

In addition, one participant working from home during the pandemic mentioned feeling healthier than before.

*(…) before Corona, I always had throat infection. I had had (inaudible) infections, pneumonia. (…) Then Corona started (…) I don’t know (if) it is because of the measures, it is because I was very careful, I don’t know, but I was healthier during Corona (…) I’m very surprised. (Healthcare worker from Eastern Africa)*

Most participants felt good about having a job during the pandemic. They mentioned worrying about the alarming situation in their home countries but feeling lucky to be in Norway.

#### Theme 3: increased perceived structural discrimination at work

Several participants mentioned they experienced discrimination at work during the pandemic, which was related to being perceived as a transmission source, meaning being infected or not vaccinated. They stated it mostly came from bosses and/or co-workers and did not arise during the pandemic. Most of those reporting increased discrimination during the pandemic had experienced discrimination in the past and referred that they believe this is a structural problem in Norwegian society.

*(…) I could see very easily, the difference when I went with my Norwegian colleague, the way they talked to him, the conversation they had, the faces they had, or the way when I went too, especially if I spoke to him in English or if I spoke to him in Norwegian, because I was just trying to speak a little bit of Norwegian (Construction worker from Southern Europe)*.

The participants stated that they perceived that not speaking the local language was also a reason for discrimination.

*If you do not speak Norwegian, they’re going to treat you completely differently. (Construction worker from Eastern Europe)*

Some mentioned that this increased discrimination also came from clients or people in the street when working outside.

*I felt… One thing that I was very, very uhmm irritating about was the skin colour because I have Somali background, then when you met the Norwegian clients they will ask are you vaccinated?, don’t come close to me. (…) after corona everything was like more, what you called it, like fear of the darkness (Healthcare worker from Eastern Africa)*.

Some participants mentioned they witnessed co-workers being discriminated against due to non-being vaccinated, but this was irrespective of being a migrant or Norwegian. Only few participants were not vaccinated, and one mentioned isolating herself because she feared discrimination.

*(…) I never went to parties or meetings that I had been invited because I was afraid they were going to ask me, are you vaccinated? so that’s why I avoided people so I practically isolated myself. (Healthcare worker from South America)*

The participants mentioned that this increased structural discrimination negatively impacted their mental health and well-being.

*(…) a person stopped and was coming down with his bicycle and told me, get out of here, you foreigner of shit, mmm you come here to infect me (with COVID-19) (…) you come here to infect us with the virus. (…) almost hit me with the bicycle so I would get out of his way (…) This really affected me a lot. (Healthcare worker from South America)*

### Host country context level

#### Theme 4: changes in social networks out of the workplace

Most participants mentioned that being far away from their family and friends in their home countries was hard, and they relied on the emotional support of their social network in Norway. The restriction measures during the pandemic made these physical encounters less frequent or not take place at all. The participants said that being unable to meet their social network due to the pandemic restriction measures negatively impacted their mental health affecting their daily life, including work.

*It was a bit hard, not being able to get together with anyone, because we ‘don’t have a family here. The only thing that makes you feel a bit welcome here are your friends, ‘isn’t it? the friends you have whether they are Norwegians, Spanish, Latin Americans or Polish. (Cleaning worker from Southern Europe)*

Some participants stated that physical contact is important for their culture. They are used to hugging and kissing friends and family. They commented that stopping doing that during the pandemic was shocking. They believe Norwegians were not affected so much by this as their culture is different and physical contact is not that common. Some participants also mentioned that virtual contact with family and friends in their home country increased during the pandemic, positively impacting their well-being.

*I think is… family is closer. I appreciate to call my family in Thailand. Before maybe some, some two or three times a week. Now it’s almost every day. (Healthcare worker from Southeast Asia)*

#### Theme 5: social protection

Most participants mentioned that they felt protected by the NAV. Only a few participants experienced job insecurity right at the beginning of the pandemic, but these fears dissipated as they realised that NAV would protect them. Some participants reported being temporarily laid off and receiving unemployment benefits from NAV. According to the participants, the social benefits in Norway worked well, making them feel grateful to live in the country during the pandemic and positively impacting their well-being. The participants also knew they could ask for sick leave when needed, whether they got COVID-19 or another physical or mental health problem during the pandemic.

*I took it (sick leave) last year in February (2022), I think it was because I was at my wits end, I couldn’t take it anymore, but it was because of the pressure from my bosses. (Healthcare worker from Southern Europe)*

Some participants also mentioned that Norwegians might consider social benefits as a right, taking for granted that these social benefits work well. However, participants that arrived as temporary workers felt more vulnerable since their access to social protection was limited as they did not have a social security number when arriving in Norway. Even if everyone, irrespective of migratory status, could be attended at the emergency room services, a social security number is necessary to access a general practitioner, the gatekeeper in the Norwegian system. According to the participants, it took some time to obtain the number. They mentioned that this negatively affected their mental and physical health as they were financially insecure and could not ask for days off if they were sick because they would not get paid for those days.

*In order to ask for time off, I had to ask for a day off work and not get paid, with the consequence that I felt that, in the eyes of my boss, I was showing an irresponsible attitude. (Transportation worker from Southern Europe)*

#### Theme 6: labour market in Norway during COVID-19

During the pandemic, temporary workers and those returning to Norway found it difficult to find new jobs. According to them, the pandemic negatively impacted the job market in Norway, and the resulting financial and job insecurity affected their mental health.

*(…) I sent a lot of CVs, they all said no and they all told me for the same reason that because of COVID nobody could promise me anything, nor did they want to hire more people because they didn’t know what the situation would be like and what it would bring. And if I was hired and they couldn’t give me a job, they had to pay me anyway. So nobody wanted to get caught. (Construction worker from Southern Europe)*

Participants from the construction sector mentioned that the lack of work put construction workers, mostly migrants, in a vulnerable position where some employers took advantage of them.

*I signed a shitty contract. The lowest an immigrant can get (…) at the worst construction site. The guy was a rat. (Construction worker from Southern Europe)*

Few participants mentioned finding better job opportunities during the pandemic and changing jobs. Moreover, two participants mentioned that they found the opportunity to open their own cleaning companies, which positively impacted their health, well-being and economic situation.

*It’s improved, it’s improved because by starting my own company, I can choose my own clients, (…).I’ve had the opportunity to buy a car to be able to go here and there, because my back was already like please stop going around with the bag all day loaded with cleaning cans, rags and the whole basket and going around with the mop in my hand in the train (…) So it has improved a lot thanks to that. (Cleaning worker from Southern Europe)*

## Discussion

In the present study, migrants working in Norway reported both mental and physical health problems related to the COVID-19 pandemic. We identified several occupation-related factors impacting positively and negatively their health and well-being: the experiences with COVID-19 measures at work, the increase in occupational hazards and perceived structural discrimination, the changes in social networks in and out of the workplace, the social benefits and changes in the labour market in Norway during the pandemic. To facilitate further research or any possible intervention by the workplace or government policymakers, we classified these factors into two levels: workplace and host country context.

Regarding the COVID-19 measures at the workplace, most of our participants reported receiving information about COVID-19 at work. Although sometimes it was too much and confusing, the overall impact on their health and well-being was positive. The importance of receiving information at work was already reported by some migrant groups in the Inncovid study conducted in Norway during the first pandemic wave [23]. Despite this, information campaigns to reach minorities in Norway have not been systematically implemented in workplaces.

Some participants reported feeling pressure at work to get vaccinated, but this was regardless of whether they were migrants. Studies have explored public opinion regarding mandatory COVID-19 vaccination in different countries, including Norway [27–30]. To our knowledge, there are no studies on COVID-19 vaccine coercion, neither on migrants nor the majority population. In Norway, as mandatory vaccination was not implemented, the fact that some workers in our study, especially healthcare workers, reported feeling pressure to get vaccinated should be further investigated as it could have legal implications [31].

Most participants reported increased psychosocial hazards during the pandemic, and few reported increased physical hazards at the workplace. The higher risk of work injuries among migrants, compared to the general population, has already been described in pre-pandemic research on working conditions and occupational health among migrants [8, 32]. During the pandemic, the research on occupational hazards among migrants is mainly related to higher rates of COVID-19 infection in essential jobs where migrants are overrepresented, such as agriculture, construction and healthcare [13]. However, there is evidence, albeit to a lesser extent, of increased psychosocial hazards for migrants during the pandemic in some specific sectors. For example, in nursing, it was reported that discrimination and racism at work were factors worsening health among migrants compared to native-born, which is consistent with our results [33]. Therefore, the worsening of other occupational hazards among migrants during the pandemic, and not only COVID-19 infections, should be explored in more detail.

Several participants felt that structural discrimination at the workplace increased during the COVID-19 pandemic and that this was related to being migrants. However, they mentioned that discrimination did not arise during the pandemic, which aligns with several studies conducted in Spain [34, 35], the United Kingdom [36, 37], and Norway [10], the latter showing systematic discrimination against migrants in the labour market [10]. Furthermore, during the pandemic, some migrant groups in vulnerable situations reported higher levels of increased discrimination [38, 39] and mental health problems among migrant workers were associated with this discrimination [38–40]. Besides discrimination, all construction workers mentioned in our interviews that they felt exploited during the pandemic. This did not emerge during the pandemic either, as exploitation of construction migrant workers and other migrant groups in Norway has long been reported [4, 41]. Moreover, in 2023, a report from the Institute of Health Equity mentioned that discrimination and exploitation are main factors for the inequality in health and well-being of migrants in Norway [4]. So, these must be urgently addressed by authorities and policymakers.

Most of the participants mentioned that unemployment in Norway increased during the first year of the pandemic, which aligns with data from Statistics Norway (SSB) and NAV [14, 42]. Study participants from the cleaning and construction sectors reported facing more problems, such as lack of work and difficulty finding jobs. They mentioned that although everyone in those sectors was affected, most of their co-workers were migrants. This is also confirmed by data from SSB showing that migrants in Norway were overrepresented in the industries most affected by the pandemic measures [42]. Several reports worldwide have confirmed that unemployment negatively impacts health through different mechanisms, such as loss of social status and fewer financial resources [4, 43, 44]. In Norway, a survey conducted periodically by SSB shows that the proportion of the unemployed population reporting good health is lower than that of the employed population reporting good health [45]. Despite this, there are no studies in Norway on how the increase in temporary layoffs affected migrants’ health and well-being during the pandemic.

Nevertheless, some other contextual factors seem to have impacted the health and well-being of migrants positively. For instance, several participants were laid off for some months at the beginning of the pandemic. Although this initially worried them, the social benefits responded adequately and positively impacted their well-being as they felt protected by the Norwegian system. This benefits system, which includes unemployment benefits, social assistance and sick pay, is generous compared to other European countries, and working migrants in legal situations are entitled to these benefits in the same way as the majority population [4]. However, this could be considered a particular factor from Norway because, according to studies in different countries, migrant workers usually have less social protection in their destination countries than the majority population [46]. Although we did not find studies in Norway on the effect of the welfare system and migrant well-being, one study found that migrants with these positive views showed more trust in the authorities in Norway [47]. On the other side, we found some exceptions in our study as temporary workers, mostly construction workers, reported some problems in accessing social benefits and asking for sick leave. These findings align with Istiko, 2022 [48], which reported that accessing social protection in Norway is harder for temporary migrant workers.

Of all the factors reported in this study, only few were considered migrant-specific. The increased structural discrimination in the workplace was the main one. Nevertheless, according to our data, the changes in social networks in and out of the workplace and the social benefits in Norway could have a differential impact on migrants’ health and well-being. Some participants mentioned the importance of their social network in Norway, which aligns with the literature reporting that social contacts and a sense of community act as a buffer in the adaptation process of migrants and reducing gaps in health and well-being between natives and migrants [49–51]. In addition, participants mentioned that Norwegians might consider social benefits as a right and take it for granted that this must be fulfilled. For migrants, the positive effect on well-being may be more pronounced as they do not always have access to good social benefits systems in their home countries. However, we have not found any literature supporting this. Positive factors should be further investigated, as research often neglects them.

Our study also has some limitations. First, a high percentage of participants were from Spanish-speaking countries, probably due to the author conducting the interviews being Spanish-speaking. In addition, interviews were performed mostly in English and Spanish, which may have resulted in overlooking other points of view and opinions from persons from other languages, even if we always offered to use an interpreter. Second, all participants were migrants in regular situations, and few were temporary workers, giving us a limited view of the experiences of groups in more vulnerable situations. Last, it was difficult for several participants to disentangle the specific consequences of the COVID-19 pandemic because they also experienced personal changes and situations that deeply affected them during this period. Despite these limitations, the main strength of our study is the recruitment of participants covering the variety of migration profiles in Norway, different working conditions, and different levels of exposure to COVID-19. Although this variety among participants does not permit work type specificities, it provides a holistic view of migrants’ experiences. Last, most of the interviews were conducted face-to-face, allowing better interaction with the participants.

The use of Braun and Clark’s systematic approach, the Benavides et al. framework, and the feedback from the rest of the research team during all phases of the analysis increased the credibility and neutrality of this study. The dependability and transferability of our research were sought by using different recruitment techniques to include participants with a variety of demographic characteristics and migration profiles and by clearly describing our sample, setting, data collection and analysis. In addition, the first author was aware of her positionality as a working migrant herself in Norway. This position helped to create a comfortable environment with the participants during the interviews, leading to them sharing more experiences and critical views. At the same time, in order to minimize bias or assumptions, we used self-reflexivity and peer-debriefing [52, 53].

## Conclusion

Our study used an occupational health framework and identified several factors affecting negatively and positively the health and well-being of migrants during the pandemic. Some of the factors were found at the workplace level, and others are part of the host country context. Workplace factors, such as pressure to get vaccinated, increased structural discrimination and exploitation, may have legal implications and should be addressed by authorities at a national level. Our study found that increased structural discrimination was the only factor clearly identified as migrant-specific by the participants, but according to them, other factors, such as the changes in social networks in and out of the workplace and social benefits, seem to have a differential impact on migrants. Further research will help us to understand better the consequences of the COVID-19 pandemic on migrants’ health and well-being. This knowledge is crucial for policymakers and authorities to design policies and develop strategies towards zero discrimination at the workplace and opening dialogue arenas for acknowledging diversity at work.

### Electronic supplementary material

Below is the link to the electronic supplementary material.

Supplementary Material 1

## Data Availability

The interview guide is included as supplementary material. The dataset is not publicly available due to privacy or ethical restrictions.

## References

[1] Shadmi ECY, Dourado I, Faran-Perach I, Furler J, Hangoma P et al. Health equity and COVID-19: global perspectives. Int J Equity Health. 2020;19(104). 10.1186/s12939-020-01218-z PMC7316580 32586388

[2] FHI. COVID-19 Ukerapport - uke 43 onsdag 3. november 2021. 2021 03-Nov-2021.

[3] FHI. Covid-19 blant innvandrere i Norge, vurdering av tiltak og erfaringer fra felt, delrapport 1. 2021 05-Jul-2021.

[4] Goldblatt P, Castedo A, Allen J, Lionello L, Bell R, Marmot M et al. Rapid review of inequalities in health and wellbeing in Norway since 2014. Norway: Institute of Health Equity; 2023 Mar 2023.

[5] Nguyen LH, Drew DA, Graham MS, Joshi AD, Guo C-G, Ma W et al. Risk of COVID-19 among front-line health-care workers and the general community: a prospective cohort study. Lancet Public Health. 2020;5. 10.1016/S2468-2667(20)30164-X PMC7491202 32745512

[6] Selden TM, Berdahl TA (2020). COVID-19 and Racial/Ethnic disparities in Health Risk, Employment, and Household Composition. Health Aff (Millwood).

[7] Reid A, Ronda-Perez E, Schenker MB. Migrant workers, essential work, and COVID-19. Am J Ind Med. 2020;1(5). 10.1002/ajim.23209 33355943

[8] Sterud T, Tynes T, Mehlum IS, Veiersted KB, Bergbom B, Airila A (2018). A systematic review of working conditions and occupational health among immigrants in Europe and Canada. BMC Public Health.

[9] Hargreaves S, Rustage K, Nellums LB, McAlpine A, Pocock N, Devakumar D (2019). Occupational health outcomes among international migrant workers: a systematic review and meta-analysis. The Lancet Global Health.

[10] Birkelund GE, Rogstad J, Heggebø K, Aspøy TM, Bjelland HF (2014). Diskriminering i arbeidslivet - resultater fra randomiserte felteksperiment i Oslo, Stavanger, Bergen og Trondheim. Sosiologisk Tidsskrift.

[11] Brekke I, Schøne P (2014). Long sickness absence differences between natives and immigrant workers: the role of differences in self-reported health. J Int Migration Integr.

[12] Tan IB, Tan C, Hsu LY, Dan YY, Aw A, Cook AR (2021). Prevalence and outcomes of SARS-CoV-2 infection among migrant workers in Singapore. JAMA.

[13] Oliva-Arocas A, Benavente P, Ronda-Perez E, Diaz E. Health of international migrant workers during the COVID-19 pandemic: a scoping review. Manuscript submitted for publication; 2021. 10.3389/fpubh.2022.816597 PMC8888537 35252094

[14] Dahl ES, Furuberg J, Helde I, Kalstø ÅM, Kann IC, Myhre A (2021). Ett år med korona. Utvikling og utsikter for NAVs ytelser og brukere, in Arbeid og velferd 1.

[15] Flølo K. Arbeidere får bare jobbe på én byggeplass. Klartale. 2021.

[16] Svendsen R, Reikerås M, Fossåskaret S. Byrådet vil stenge ned store byggeplasser i Bergen. Bransjen får svar i kveld. NRK. 2021 09-Feb-2021.

[17] Hayward SE, Deal A, Cheng C, Crawshaw A, Orcutt M, Vandrevala TF (2021). Clinical outcomes and risk factors for COVID-19 among migrant populations in high-income countries: a systematic review. J Migr Health.

[18] FHI. Smitterisiko og konsekvenser av covid-19 blant personer med innvandrerbakgrunn: en hurtigoversikt. 2021 24-Jun-2021.

[19] Tong A, Sainsbury P, Craig J (2007). Consolidated criteria for reporting qualitative research (COREQ): a 32-item checklist for interviews and focus groups. Int J Qual Health Care.

[20] NAV. Information about NAV’s services and benefits 2023 [Available from: https://www.nav.no/en/home/benefits-and-services/information-about-nav-s-services-and-benefits.

[21] SSB. Labour force survey. 2023.

[22] Inncovid.Norge. Inncovid.Norge Norway2020 [Available from: https://www.inncovid.no/en/.

[23] Madar AA, Benavente P, Czapka E, Herrero-Arias R, Haj-Younes J, Hasha W et al. COVID-19: access to information, level of trust and adherence to health advice among migrants in Norway. Archives of Public Health. 2022;80(15). 10.1186/s13690-021-00764-4 PMC8725426 34983639

[24] WHO, ApartTogether Survey. WHO; 2020 2020.

[25] Braun V, Clarke V (2006). Using thematic analysis in psychology. Qualitative Res Psychol.

[26] Ruiz-Frutos C, Delclòs J, Ronda E, García A, Benavides F. Salud laboral: conceptos y tecnicas para la prevencion de riesgos laborales (Occupational Health: concepts and techniques for peventing occupational risks). 5th ed. Barcelona: Elsevier; 2022 2022.

[27] Gur-Arie R, Jamrozik E, Kingori P (2021). No jab, no job? Ethical issues in mandatory COVID-19 vaccination of Healthcare Personnel. BMJ Global Health.

[28] King J, Ferraz OLM, Jones A (2022). Mandatory COVID-19 vaccination and human rights. The Lancet.

[29] Maneze D, Salamonson Y, Grollman M, Montayre J, Ramjan L. Mandatory COVID-19 vaccination for healthcare workers: A discussion paper. International Journal of Nursing Studies. 2023;138:104389. 10.1016/j.ijnurstu.2022.104389 PMC9709452 36462385

[30] Schmelz K, Bowles S. Opposition to voluntary and mandated COVID-19 vaccination as a dynamic process: Evidence and policy implications of changing beliefs. Proceedings of the National Academy of Sciences. 2022;119(13):e2118721119. 10.1073/pnas.2118721119 PMC9060490 35316133

[31] Holmøyvik E, Lex-Atlas. Covid-19 [Internet]. UK2021. [cited 2023 27-Mar-2023]. Available from: https://lexatlas-c19.org/norway-voluntary-vaccination-is-the-policy-law-allows-for-more-but-probably-not-now/.

[32] Al-Maskari F, Shah SM, Al-Sharhan R, Al-Haj E, Al-Kaabi K, Khonji D (2011). Prevalence of Depression and suicidal behaviors among male migrant workers in United Arab Emirates. J Immigr Minor Health.

[33] Llop-Gironés A, Vračar A, Llop-Gironés G, Benach J, Angeli-Silva L, Jaimez L (2021). Employment and working conditions of nurses: where and how health inequalities have increased during the COVID-19 pandemic?. Hum Resour Health.

[34] Diaz-Serrano L (2013). Immigrants, natives and job quality: evidence from Spain. Int J Manpow.

[35] Gil-González D, Vives-Cases C, Borrell C, Agudelo-Suárez AA, Davó-Blanes MC, Miralles J (2014). Racism, other Discriminations and Effects on Health. J Immigr Minor Health.

[36] Bhui K, Stansfeld S, McKenzie K, Karlsen S, Nazroo J, Weich S (2005). Racial/ethnic discrimination and common mental disorders among workers: findings from the EMPIRIC study of ethnic minority groups in the United Kingdom. Am J Public Health.

[37] Wadsworth E, Dhillon K, Shaw C, Bhui K, Stansfeld S, Smith A (2006). Racial discrimination, ethnicity and work stress. Occup Med.

[38] Spiritus-Beerden E, Verelst A, Devlieger I, Langer Primdahl N, Botelho Guedes F, Chiarenza A (2021). Mental Health of Refugees and Migrants during the COVID-19 pandemic: the role of experienced discrimination and daily stressors. Int J Environ Res Public Health.

[39] Song J, McDonald C (2021). Experiences of New Zealand registered nurses of chinese ethnicity during the COVID-19 pandemic. J Clin Nurs.

[40] Zhang M, Gurung A, Anglewicz P, Baniya K, Yun K (2022). Discrimination and stress among Asian Refugee populations during the COVID-19 pandemic: evidence from bhutanese and burmese Refugees in the USA. J Racial Ethn Health Disparities.

[41] Brunovskis A, Ødegård A. Grov utnytting av utenlandske arbeidstakere - Gråsonen mellom det regulære arbeidslivet og menneskehandel (Gross exploitation of migrant workers - The grey zone between the regular labour market and human trafficking). Oslo: Fafo; 2022 2022.

[42] Olsen B (2022). Innvandrere var mest utsatt på arbeidsmarkedet under pandemien (immigrants were the mostvulnerable on the labormarket during the pandemic).

[43] Marmot M, Goldblatt P. J. A. Fair Society healthy lives (the Marmot Review). Institute of Health Equity; 2010.

[44] Eliason M, Storrie D (2009). Job loss is bad for your health – swedish evidence on cause-specific hospitalization following involuntary job loss. Soc Sci Med.

[45] STAMI (2021). Faktabok om arbeidsmiljø og helse (Factbook on working environment and health).

[46] Bossavie LLY, Garrote Sanchez D, Makovec M, Ozden C, Occupational Hazards (2021). Why Migrants Faced Greater Economic and Health Risks during the COVID-19 pandemic (English).

[47] Herrero-Arias R, Ortiz-Barreda G, Czapka E, Diaz E (2022). The evolvement of trust in response to the COVID-19 pandemic among migrants in Norway. Int J Equity Health.

[48] Istiko SN, Durham J, Elliott L. (Not that) essential: a scoping review of migrant workers’ Access to Health Services and Social Protection during the COVID-19 pandemic in Australia, Canada, and New Zealand. Int J Environ Res Public Health. 2022;19(5). 10.3390/ijerph19052981 PMC8909973 35270672

[49] Arpino B, de Valk H (2018). Comparing life satisfaction of immigrants and natives across Europe: the role of Social Contacts. Soc Indic Res.

[50] Hombrados-Mendieta MI, Gomez-Jacinto L, Dominguez-Fuentes JM, Garcia-Leiva P, SENSE OF COMMUNITY, AND SATISFACTION WITH LIFE AMONG IMMIGRANTS AND THE NATIVE POPULATION (2013). J Community Psychol.

[51] Adedeji A (2021). Social Capital and Migrants’ quality of life: a systematic narrative review. J Int Migration Integr.

[52] Ide Y, Beddoe L. Challenging perspectives: Reflexivity as a critical approach to qualitative social work research. Qualitative Social Work.0(0):14733250231173522.

[53] Janesick VJ. Peer debriefing. The Blackwell Encyclopedia of Sociology.

