# Supplementary material for: Occupation-related factors affecting the health of migrants working during the COVID-19 pandemic – a qualitative study in Norway
Source: Int J Equity Health. 2023 Oct 17;22:220. doi: 10.1186/s12939-023-02013-2 (PMC10583456; doi:10.1186/s12939-023-02013-2)
Supplement: Supplementary file 1 — Supplementary Material 1 [file 12939_2023_2013_MOESM1_ESM.docx]

INTRO *(turn the recorder on)*

1. *(Greetings, breaking the ice)*
2. Have you received the consent form to participate in this project? Do you have any questions regarding the project?
3. I am performing this st udy to gather information from working migrants from different types of jobs. I want to understand how they have managed the pandemic and the vaccination process, the consequences of COVID-19 and its measures, and also, why do you think there are more COVID-19 cases among migrants than among general population. We are recording this conversation. Is that ok for you?

SOCIO-DEMOGRAPHIC QUESTIONS

Could you let me know a little bit about yourself?

1. How old are you?
2. Where do you come from, and for how long time have you been in Norway?
   1. Have you come only for a limited period of time?
3. Who do you live with in Norway? Are they Norwegian? What kind of contact do you have with Norwegians?
4. What do you do for a living? Are you working now? What do you do at work?
5. Which language do you speak at home? And at work?

QUESTIONS REGARDING PANDEMIC

*Regarding pandemic in general:*

1. The pandemic is not over yet; after more than two years, how have your life been affected by the pandemic and the mitigation measures?

*Probes*

- 1. Did anybody around you got infected by the COVID?
  2. What in your life has changed most in relation to the Covid pandemic?
  3. Any other areas that have been changed (your work, your situation as a migrant) either because of the pandemic or the measures?
  4. Any other thing related to your health?
  5. Anything that has improved? Could you explain briefly how?

Did any of these experiences change during the different stages of the pandemic?

*Regarding your work and the pandemic:*

1. How have been your experience as a working migrant during the pandemic? (Probing: exposure to COVID and to other occupational risks, job stability, support from employees, commuting to work, any change in your work, work productivity)
2. Do you know any person in your family or your migrant group that has had the same experiences with their jobs? If so, how?

*Regarding positive factors during the pandemic:*

1. Is there something/someone (factors) that has helped you/your household/your migrant group during this pandemic period?
   1. Can you explain briefly how?
2. Do members of your household or your migrant community have had similar or different experiences? Can you give examples of how? Any other examples? Anything that has improved for them?

*General situation and discrimination*

1. Although the situation in different migrant groups is not the same, in general migrants have a higher risk of being infected and hospitalized due to COVID-19 worldwide, including Norway. Why do you think this happens?
   1. What do you mean by (any factor they can mention)? Can you give an example?
2. During the pandemic, some migrant groups from different countries, such as migrants from Southeast Asia (other groups if the migrant is from this region), have experienced some discrimination. Have you heard about this? What have you heard? Have you experienced something similar linked to COVID-19 and being a migrant? If so, could you tell a bit about these experiences?

*Regarding vaccination:*

1. Now, can I ask you about vaccination? If *Yes*, are you vaccinated? *If no*, are you planning to get vaccinated?
2. How do you come to that decision? What influenced your decision?
3. *If participant wish to be vaccinated, how easy it is/was? Why? (probe on barries and facilitators) Did you get the offer to get vaccinated?*
4. Has your employer provided you with information about vaccination or has facilitated getting the shot? How?
5. Are people in your household or migrant group have had the same experiences with vaccination? Could you give some examples?
6. Is there something else you want to share with me?
